# Prevalence of vaping, vaping-associated short-term symptoms of respiratory and cardiovascular morbidities, and factors associated with the initiation of vaping among young adults in Kuwait

**DOI:** 10.18332/tid/201441

**Published:** 2025-03-19

**Authors:** Aisha Altanaib, Arwa Alkhannah, Dhuha Alfouderi, Mariam Almutairi, Rawan Abdullah, Maryam Almuhaileej, Fajer Alqadeeri, Reema Alajmi, Loulwah Alenizi, Ahmad Alsultan, Saeed Akhtar

**Affiliations:** 1 Department of Community Medicine and Behavioral Sciences, College of Medicine, Kuwait University, Safat, Kuwait

**Keywords:** vaping, risk factors, young adults, respiratory and cardiovascular morbidity

## Abstract

**INTRODUCTION:**

E-cigarette use or vaping is a public health concern, especially among young adults worldwide. This cross-sectional study aimed to: 1) assess the prevalence of vaping among young adults; 2) assess the prevalence of short-term vaping-associated respiratory and cardiovascular symptoms; and 3) identify factors associated with vaping status among a student population in Kuwait.

**METHODS:**

In October 2024, a cross-sectional study enrolled students, aged ≥18 years, from various colleges of Kuwait University. Data were collected using a structured e-questionnaire administered through in-person invitations and online platforms. The prevalence (%) of vaping was computed. A multivariable log-binomial regression model was used to estimate adjusted prevalence ratios (APRs) and corresponding 95% confidence intervals (CI) for the factors significantly associated with vaping status. All the statistical tests were two-tailed.

**RESULTS:**

Of 1144 participants, most were females (78.5%), Kuwaiti (85.5%), and aged 18–21 years (70.6%). The prevalence of vaping in our sample was 15.5% (177/1144). After adjusting for the effects of age and monthly family income (in KWD), the factors that were significantly (p<0.05) and independently associated with vaping status were male gender (APR=4.52; 95% CI: 3.28–6.22), being a student at a literary college (APR=1.50; 95% CI: 1.12–2.02), a positive belief that ‘vaping is less harmful than cigarette smoking’ (APR=1.46; 95% CI: 1.06–2.01), and a ‘disbelief that vaping leads to cigarette smoking’ (APR=1.80; 95% CI: 1.32–2.45), and ‘perception about easy accessibility of vaping products’ (APR=3.27; 95% CI: 1.04–10.32).

**CONCLUSIONS:**

A moderately high prevalence (15.5%) of vaping in the study sample was recorded. Male gender and some misplaced beliefs and perceptions were significantly associated with vaping status in the study sample. The high prevalences of respiratory symptoms among the participants call for targeted interventions. If instituted, future studies may evaluate the impact of such efforts.

## INTRODUCTION

E-cigarette use or vaping is an emerging public health problem with increasing popularity in various populations worldwide, especially among young adults^1^. Vaping may serve as a new gateway for nicotine addiction with serious cardio-pulmonary complications^2^. Nicotine, a major component of e-cigarettes, is a stimulant of the sympathetic nervous system that is associated with several short-term cardiovascular and hemodynamic side effects. It results in an increased heart rate, elevated blood pressure, and amplified myocardial contractility^3^. Moreover, the aerosolized glycol mixtures found in e-cigarettes have an association with various respiratory ailments. For instance, e-cigarette consumers reportedly experience short-term symptoms, such as dry cough, throat irritation, and reduced lung function, particularly among individuals with higher levels of exposure^4^. The dual use of tobacco cigarettes and e-cigarettes leads to an increased risk of developing chronic cough, phlegm, bronchitis, and dyspnea owing to endothelial dysfunction, increased platelet activation, aggregation, oxidative stress, and inflammatory reactions^5,6^.

The prevailing misconception that vaping is safer than traditional cigarette smoking is a critical factor in the widespread use of e-cigarettes among young adults, including people who never smoked cigarettes^7^. The use of e-cigarettes among young adults who have never smoked previously not only exposes themselves to vaping-related health risks, but also they tend to start smoking traditional cigarettes^1,8^. Another important element that contributes to the popularity of vaping is the increasing number of smokers who are intending to reduce or quit combustible cigarettes and are turning to e-cigarettes, considering them safer^2^. In contrast, the results of a meta-analysis revealed insufficient evidence regarding the effectiveness of e-cigarette use for short- and/or long-term cessation of cigarette smoking^9^.

E-cigarette usage has become a significant public health threat due to its increasing spread worldwide, with an estimated 58.1 million users in 2018 and 68 million in 2020^1,10^. Among youth (aged 12–16 years), the global prevalence of past-30-day e-cigarette use is 9.8%^11^. In Middle Eastern countries, the estimated prevalence of current e-cigarette consumption is mainly based on the assessment among university students, including UAE (23% to 25%)^9,12^, Saudi Arabia (27.7%)^13^, and Qatar (14%)^14^. E-cigarette use has rapidly increased among Kuwait’s population in which the prevalence of ever e-cigarette use among adults is 40.2%, and current (past 30-day) consumption is 29.4%. Furthermore, in Kuwait, e-cigarette use is more common among males (47.6%) than females (14.4%)^15^. Another study revealed that 26.4% of high school students reported to be current e-cigarette users^16^. The alarming trend of vaping among adolescents in Kuwait is particularly concerning as it increases the chances of early nicotine addiction and future health complications.

To address this challenge of vaping and tobacco use through other modes, Kuwait has implemented several laws and policies to address this problem. In 2006, Kuwait ratified the World Health Organization Framework Convention on Tobacco Control (WHO FCTC) and enforced multiple measures to limit the usage of tobacco products. The government banned smoking in public places, workplaces, and public transportation, and prohibited the advertisement of tobacco products. Moreover, the government mandated the placement of warning labels on tobacco product packages, raised tobacco product prices, and raised the minimum age for purchasing tobacco products from 18 to 21 years^15,16^. Despite these efforts, e-cigarette use continued to be a growing major public health concern, mainly in the young population. There are currently no policies to limit and minimize e-cigarette consumption in Kuwait other than the age limit. Furthermore, there is limited published data on the prevalence of vaping and the risk factors that contribute to the induction of vaping among young adults in Kuwait. Therefore, this cross-sectional study was designed to: 1) assess the prevalence of vaping among young adults; 2) assess the prevalence of the self-reported short-term vaping-associated respiratory and cardiovascular symptoms in the study population; and 3) identify factors associated with the initiation of vaping in the study population. The results of this study are likely to gauge the magnitude of the problem in the young population and identify risk factors that may furnish the basis for designing and implementing a prevention program to minimize the use of e-cigarettes.

## METHODS

### Study design, setting, and participants

This cross-sectional study was carried out in October 2024. For this purpose, the participants aged ≥18 years were enrolled from various colleges of Kuwait University, a public sector premier institution for undergraduate and graduate studies. Kuwait University has 17 colleges located on two different campuses with a total enrolment of 39891 students during the 2024–2025 academic year. All the students enrolled in Kuwait University were eligible for participation in this study. Invitations to participate were made face-to-face, and an e-questionnaire was shared with consenting participants through a social media platform, i.e. WhatsApp, and participants were encouraged to spread them to their colleagues. Hence, the snowball sampling technique – a non-probability sampling method – was used as a convenience sampling method.

### Questionnaire

An e-questionnaire was developed in English and Arabic for data collection. The questions were designed based on the relevant literature review^17,18^, and comprised 31 questions, which were grouped into four sections, including: 1) sociodemographics; and 2) vaping practices; particularly, the participant was asked ‘Have you ever used a vape (or e-cigarette). The participants with positive responses (i.e. ‘yes’) were further inquired about the frequency of vape use, age (years) at the initiation of vaping, and duration (years) of vaping; 3) factors potentially associated with vaping; and 4) resultant ill-health symptoms potentially associated with vaping. The questionnaire was reviewed by two faculty members of the Department of Community Medicine and Behavioral Sciences, College of Medicine. The necessary adjustments were made as indicated.

### Sampling, and data collection

The data were collected by three data collection teams, each comprising three 5th-year medical students. The students were selected as a sample of convenience from various colleges of Kuwait University. A structured, self-administered e-questionnaire was created as a Google Form. Between 13 and 17 October 2024, data collection teams visited the premises of assigned colleges and met the students at the end of their classes or in cafeterias. The data collection teams explained the study’s objectives to the students and requested their participation. The team members shared the e-questionnaire (Google Form) with the students who consented. The informed consent was on the first page of the e-questionnaire, and respondents were requested to read and sign it before answering the questions. We checked the filled-in questionnaire on the spot to verify its completion. Furthermore, the methods, including design, conduct, data collection, analysis, and reporting of the results, conformed with the STROBE (Strengthening the Reporting of Observational Studies in Epidemiology) guidelines (Supplementary file Table 1).

### Ethics

The study protocol and the study instrument were approved by the Kuwait University Health Sciences Center Ethics Committee (no. 794/ Dated: 03/10/2024). Informed written consent was obtained from each of the participants. The participants were assured of the confidentiality of the collected information. The data collection was anonymous, as no personal identification information was collected or was a part of the database. This study was undertaken by the principles and guidelines of the Declaration of Helsinki for medical research involving human subjects. A formal permission was sought from the vice deans for academics of the colleges of university. It was inappropriate or impossible to involve patients or the public in the design, conduct, reporting, or dissemination plans of our research.

### Data analysis

Data were gathered using Google Forms, converted to a Microsoft Excel file, and then transferred into the SPSS (IBM Statistical Package for Social Sciences Ver 29.0.1.1) data file. The data file was reviewed and cleaned to exclude errors and inadmissible entries before statistical analysis. Descriptive statistics of sociodemographics, including proportions (%) of categorical variables, were computed to characterize the study sample. The outcome variable, vaping status, was defined as an affirmative case of vaping (coded=1) and negative otherwise (coded=0). The prevalence (%) of vaping and the self-reported symptoms were computed. As appropriate, chi-squared analysis or chi-squared analysis for trend for the ordinal independent variable was carried out to examine the statistical associations of demographics and risk factors with vaping status. Univariable log-binomial regression analysis was conducted to quantify the association of the variables significantly (p≤0.250) related to the vaping status on the chi-squared analysis and were considered for probable inclusion in the multivariable model. To identify the variables independently and significantly (p<0.05) associated with vaping status, a multivariable log-binomial regression model was fitted to the data. A backward stepwise procedure was used to arrive at the final model, and the variables significantly (p<0.05) and independently associated with the vaping status were retained in the model. Adjusted prevalence ratios (APRs) and their corresponding 95% confidence intervals (CI) were used to interpret the model.

For this cross-sectional study, we estimated a sample size of 984 participants to assess the prevalence of self-reported vaping (e-cigarette use) at 95% confidence level (1-α) with 5% bound on the error of estimation assuming a prevalence of self-reported hypertension as 20% in our target population^19^. To account for refusals, the design effect of 1, and the number of age-sex groups for which estimates were intended to compute, the sample size was increased to 1100 participants. Furthermore, this sample size was large enough to relate most of the potential exposures (with a prevalence of 0.25 or higher in the population at-large) with the outcome.

## RESULTS

### Study sample, prevalence of vaping and related self-reported short-term symptoms

A total of 1165 Kuwait University students were invited, and 1144 (98.2 % response rate) consented and participated in this cross-sectional study, of whom 898 (78.5%) were females. The participants who refused (1.8%) to participate cited being busy as the reason. The participants were predominantly aged between 18 and 21 years (70.6%). Moreover, most of the participants were Kuwaiti nationals (85.5%) and residents of either Farwaniya (21.4%) or Capital (20.5%). In addition, most students were enrolled in scientific colleges (64.5%) and were in academic years 1–3 (65.7%). Of the participants, 57.9% reported that their monthly family income (KWD) was >1500, and 28.2% reported their monthly family income in the range of 1000–1500 (Table 1).

**Table 1:** Sociodemographics and vaping characteristics of participants, a cross-sectional study among young adults in Kuwait, October 2024 (N=1144)

*Characteristics*	*n*	*%*
**Age** (years)		
18–21	808	70.6
>21	336	29.4
**Gender**		
Female	898	78.5
Male	246	21.5
**Nationality**		
Kuwaiti	978	85.5
Non-Kuwaiti	166	14.5
**Academic year**		
1–3	752	65.7
4–7	392	34.3
**Governorate of residence**		
Capital	234	20.5
Ahmadi	196	17.1
Hawalli	214	18.7
Jahra	143	12.5
Mubarak Alkabeer	112	9.8
Farwaniya	245	21.4
College of enrolment*		
Literary	406	35.5
Science	738	64.5
**Monthly family income** (KWD)		
<500	51	4.5
500–999	108	9.4
1000–1500	323	28.2
>1500	662	57.9
**Vaping status**		
Yes	177	15.5
No	967	84.5
**Frequency of vaping**		
Daily	101	57.1
Weekly	9	5.1
Monthly	3	1.7
Rarely	64	36.2
**Average vaping sessions per day**		
1–3	68	38.4
4–6	15	8.5
≥7	94	53.1
**Age of first vaping** (years)		
<12	11	6.2
12–14	17	9.6
15–17	74	41.8
18–21	61	34.5
>21	14	7.9
**Length of time of vaping** (years)		
<1	41	23.2
1–2	34	19.2
>2	102	57.6
**Type of vaping product**		
Disposable e-cigarettes	112	63.6
Refillable e-cigarettes	28	15.9
Vape pens	22	12.5
Nicotine-free e-liquids	4	2.3
All of the above	4	2.3
Other	6	3.4
**Motivation to start vaping**		
Peer influence	30	16.9
Curiosity	105	59.3
Stress relief	5	2.8
To quit smoking cigarettes	26	14.7
Flavor options	8	4.5
Other	3	1.7
**Source of introduction to vaping**		
Friends	114	64.4
Family	21	11.9
Social media	23	13
Retailers	9	5.1
Myself	8	4.5
All of the above	2	1.1
**Influence of friends on decision to vape**		
Very influential	41	23.2
Somewhat influential	73	41.2
Not influential	63	35.6
**Likelihood of vaping becoming a long-term habit**		
Very likely	58	32.8
Somewhat likely	42	23.7
Not very likely	32	18.1
Not at all likely	45	25.4
**Tried to quit vaping**		
Yes	141	79.7
No	36	20.3

* Literary: College of Law, Business Administration, Arts, Education, Shari and Islamic Studies, Social Sciences. Science: College of Medicine, Public Health, Dentistry, Pharmacy, Allied Health Sciences, Life Sciences, Sciences, Engineering and Petroleum, Architecture. KWD: 1 Kuwaiti Dinar about US$3.24.

The characteristics of vapers are also shown in Table 1. The prevalence of vaping (e-cigarette use) in the study sample was 15.5% (177/1144). Among the 177 vapers, reportedly 57.1% were daily vape users, and 42.4% were using these products more than ten times per day. Most vapers (41.8%) initiated vaping when they were aged 15–17 years, and 57.6% of the vapers had been using these products for more than two years. Of the vapers, most (63.6%) use disposable e-cigarettes. Curiosity was the dominant (59.3%) motivation for vapers to start vaping, and the predominant source of introduction to vaping was friends (64.4%). When asked about the likelihood of vaping becoming a long-term habit, 32.8% indicated that it was ‘very likely’. A significant majority (79.7%) had attempted to quit vaping. Self-reported symptoms among vapers and non-vapors in the sample are presented in Table 2. The self-reported short-term vaping-related respiratory and cardiovascular symptoms were significantly (p<0.05) more common among vapors than non-vapors for cough (52.0% vs 41.0%), dry mouth (56.5% vs 29.5%), chest pain (46.9% vs 30.8%), palpitations (50.3% vs 34.4%) and shortness of breath (62.7% vs 37.4). However, more non-vapers than vapors (23.9% vs 15.8%) sought medical attention for their symptoms.

**Table 2:** Relative prevalence of short-term symptoms of respiratory and cardiovascular morbidities, a cross-sectional study among young adults in Kuwait, October 2024 (N=1144)

*Variable*	*Vapers*		*Non-vapers*		*p*
	*n*	*%*	*n*	*%*	
**Total**	177	15.5	967	84.5	
**Experienced symptoms** (yes vs no)					
Sore throat	64	36.2	353	36.5	0.930
Cough	92	52.0	369	41.0	**0.006**
Dry mouth	100	56.5	285	29.5	**<0.001**
Chest pain	83	46.9	298	30.8	**<0.001**
Palpitations	89	50.3	333	34.4	**<0.001**
Shortness of breath	111	62.7	362	37.4	**<0.001**
Sought medical attention for vaping-related symptoms	28	15.8	231	23.9	0.018

### Factors associated with vaping status

The results of the chi-squared analysis showed that the sociodemographics that were significantly (p<0.05) associated with the vaping status included age, gender, college of study, and monthly family income (KWD). Of the vaping-related factors, various perceptions, beliefs, and influences that prompted the initiation of vaping that were significantly associated with vaping status are given in Table 3. The unadjusted prevalence ratios (PRs) for the variables significantly (p≤0.250) related to vaping status by chi-squared analysis are presented in Table 4. The participants aged >21 years had a 1.40 times higher prevalence of vaping compared with those aged 18–21 years (PR=1.33; 95% CI: 1.00–1.96, p=0.048). Compared with female participants, males were significantly more likely to be the current vapors (PR=5.85; 95% CI: 4.48–7.65, p<0.001). Students enrolled in literary colleges had a 1.68 times higher prevalence of vaping than those in scientific colleges (PR=1.68; 95% CI: 1.28–2.20, p<0.001). The participants who believed that ‘vaping is less harmful than smoking’ were significantly more likely to be the vapors (PR=1.46; 95% CI: 1.06–2.01; p=0.019). PRs for other vaping-related beliefs and perceptions of the current vaping status are also given in Table 4.

**Table 3:** Chi-squared analysis of the factors associated with vaping status, a cross-sectional study among young adults in Kuwait, October 2024 (N=1144)

*Factors*	*Vaping*	*No vaping*	*p*
	*n (%)*	*n (%)*	
**Total**	177 (15.5)	967 (84.5)	
**Sociodemographic**			
**Age** (years)			0.048
18–21	114 (14.1)	694 (85.9)	
>21	63 (18.8)	273 (81.3)	
**Gender**			<0.001
Female	68 (7.6)	830 (92.4)	
Male	109 (44.3)	137 (55.7)	
**Nationality**			0.441
Kuwaiti	148 (15.1)	830 (84.9)	
Non-Kuwaiti	29 (17.5)	137 (82.5)	
**College of study**			<0.001
Literary	85 (20.9)	321 (79.1)	
Scientific	92 (12.5)	646 (87.5)	
**Academic year**			0.150
1–3	108 (14.4)	644 (85.6)	
4–7	69 (17.6)	323 (82.4)	
**Governorate**			0.435
Capital	39 (16.7)	195 (83.3)	
Ahmadi	34 (17.3)	162 (82.7)	
Hawalli	32 (15)	182 (85)	
Jahra	14 (9.8)	129 (90.2)	
Mubarak Alkabeer	20 (17.9)	92 (82.1)	
Farwaniya	38 (15.5)	207 (84.5)	
**Monthly family income** (KWD)			0.022^a^
>1500	105 (15.9)	557 (84.1)	
1000–1500	41 (12.7)	282 (87.3)	
500–999	16 (14.8)	92 (85.2)	
<500	15 (29.4)	36 (70.6)	
**Vaping-related**			
**Belief that vaping is less harmful than smoking**			<0.001
Yes	62 (27.9)	160 (72.1)	
No/not sure	115 (12.5)	807 (87.5)	
**Belief that vaping leads to smoking cigarettes**			<0.001
No/not sure	70 (26.82)	191 (73.18)	
Yes	107 (12.12)	776 (87.88)	
**Perception of vaping-related health risks compared with smoking**			<0.001
Lower	57 (23.6)	185 (76.4)	
Same	68 (12.8)	464 (87.2)	
Higher	52 (14.1)	318 (85.9)	
**Exposure to advertisements or promotional material about vaping**			0.544
No	66 (14.7)	384 (85.3)	
Yes	111 (16.0)	583 (84.0)	
**Pressured by peers to vape**			<0.001
Yes	49 (26.5)	136 (73.5)	
No	128 (13.3)	831 (86.7)	
**Advertisements’ influence on the decision to initiate vaping**			<0.001
Very influential	16 (29.1)	39 (70.9)	
Somewhat influential	16 (18.6)	70 (81.4)	
Not very influential	41 (28.3)	104 (71.7)	
Not at all influential	48 (9.7)	446 (90.3)	
Haven’t seen any advertisements	56 (15.4)	308 (84.6)	
**Knowledge of the health risks associated with vaping**			0.115
Very knowledgeable	95 (17.6)	444 (82.4)	
Somewhat knowledgeable	71 (14.1)	432 (85.9)	
Not knowledgeable	11 (10.8)	91 (89.2)	
**Perception of availability of vaping products in area**			<0.001
Very accessible	149 (19.0)	635 (81.0)	
Somewhat accessible	25 (8.4)	273 (91.6)	
Not accessible	3 (4.8)	59 (95.2)	
**Opinion on university policies regarding vaping on campus**			0.465
Supportive	73 (16.9)	360 (83.1)	
Neutral	71 (15.3)	392 (84.7)	
Opposed	33 (13.3)	215 (86.7)	

a P-value of chi-squared test statistic for trend.

* Literary: College of Law, Business Administration, Arts, Education, Shari and Islamic Studies, Social Sciences. Science: College of Medicine, Public Health, Dentistry, Pharmacy, Allied Health Sciences, Life Sciences, Sciences, Engineering and Petroleum, Architecture. KWD: 1 Kuwaiti Dinar about US$3.24.

**Table 4:** Univariable log-binomial regression analysis of the factors associated with vaping status, a cross-sectional study among young adults in Kuwait, October 2024 (N=1144)

*Factors*	PR*	*95% CI*	*p*
**Sociodemographic**			
**Age** (years)			0.048
18–21 ®	1		
>21	1.33	1.00–1.76	
**Gender**			**<0.001**
Female ®	1		
Male	5.85	4.48–7.65	
**College of study**			**<0.001**
Scientific ®	1		
Literary	1.68	1.28–2.20	
**Academic year**			0.151
1–3 ®	1		
4–7	1.23	0.93–1.62	
**Monthly family income** (KWD)			**0.028**
>1500 ®	1		
1000–1500	0.80	0.57–1.12	
500–999	0.93	0.57–1.52	
<500	1.85	1.17–2.94	
**Vaping-related**			
**Belief that vaping is less harmful than smoking**			**<0.001**
No/not sure ®	1		
Yes	2.24	1.70–2.95	
**Belief that vaping leads to smoking cigarettes**			**<0.001**
Yes ®	1		
No/not sure	2.21	1.69–2.89	
**Perception of vaping-related health risks compared with smoking**			**<0.001**
Higher ®	1		
Lower	1.68	1.19–2.35	
Same	0.91	0.65–1.27	
**Pressured by peers to vape**			**<0.001**
No ®	1		
Yes	1.98	1.49–2.65	
**Advertisements’ influence on the decision to initiate vaping**			**<0.001**
Haven’t seen any advertisements ®	1		
Very influential	2.13	1.37–3.31	
Somewhat influential	1.21	0.73–2.00	
Not very influential	1.84	1.29–2.62	
Not at all influential	0.63	0.44–0.91	
**Knowledge of the health risks associated with vaping**			0.115
Very knowledgeable ®	1		
Somewhat knowledgeable	0.80	0.60–1.06	
Not knowledgeable	0.61	0.34–1.10	
**Perception of availability of vaping products in area**			**<0.001**
Not accessible ®	1		
Somewhat accessible	1.73	0.54–5.56	
Very accessible	3.93	1.29–11.96	

* PR: unadjusted prevalence ratio. Literary: College of Law, Business Administration, Arts, Education, Shari and Islamic Studies, Social Sciences. Science: College of Medicine, Public Health, Dentistry, Pharmacy, Allied Health Sciences, Life Sciences, Sciences, Engineering and Petroleum, Architecture. KWD: 1 Kuwaiti Dinar about US$3.24. ® Reference categories.

### Multivariable log-binomial regression model

The final multivariable log-binomial regression model of the factors significantly and independently associated with vaping status in the study sample shows that after adjustment for the monthly family income (KWD), compared with the females, the male participants were significantly more likely tended to be current vapers (APR=4.52; 95% CI: 3.28–6.22; p<0.001) or enrolled in a literary rather than scientific college (APR=1.50; 95% CI: 1.12–2.02; p=0.007). Furthermore, the participants were significantly more likely to be vapers if they believed that ‘vaping is less harmful than smoking’ (APR= 1.46; 95% CI: 1.06–2.01; p=0.019), disbelieved/uncertain that ‘vaping leads to smoking’ (APR=1.80; 95% CI: 1.32– 2.45; p<0.001), or believed that ‘vaping products are very accessible’ (APR=3.27; 95% CI: 1.04–10.32; p=0.043) compared with the participants in respective reference categories of these factors (Table 5).

**Table 5:** Multivariable log-binomial regression model of the factors associated with vaping status, a cross-sectional study among young adults in Kuwait, October 2024 (N=1144)

*Factors*	APR*	*95% CI*	*p*
**Gender**			**<0.001**
Female ®	1		
Male	4.52	3.28–6.22	
**College of study**			
Scientific ®	1		
Literary	1.50	1.12–2.02	**0.007**
**Belief that vaping is less harmful than smoking**			**0.019**
No/not sure ®	1		
Yes	1.46	1.06–2.01	
**Belief that vaping leads to smoking cigarettes**			**<0.001**
Yes ®	1		
No/not sure	1.80	1.32–2.45	
**Perception of availability of vaping products in area**			
Not accessible ®	1		0.365
Somewhat accessible	1.74	0.52–5.77	**0.043**
Very accessible	3.27	1.04–10.32	
**Monthly family income** (KWD)			
>1500 ®	1		
1000–1500	1.15	0.80–1.66	0.466
500–999	1.06	0.62–1.80	0.823
<500	1.97	1.14–3.40	**0.015**

* APR: adjusted prevalence ratio. Literary: College of Law, Business Administration, Arts, Education, Shari and Islamic Studies, Social Sciences. Science: College of Medicine, Public Health, Dentistry, Pharmacy, Allied Health Sciences, Life Sciences, Sciences, Engineering and Petroleum, Architecture. KWD: 1 Kuwaiti Dinar about US$3.24. ® Reference categories.

## DISCUSSION

This cross-sectional study reports the prevalence of e-cigarette usage in a sample of Kuwait university students, and examines the association of sociodemographics and risk factors with vaping status. In this study, the prevalence of current e-cigarette use was 15.5%. This estimate of vaping is comparable with an estimate (14%) among university students in Qatar^14^. However, compared with the prevalence of vaping in the present study, some other countries in the region reported a higher prevalence of vaping among university students, including Saudi Arabia (27.7%)^13^, UAE (23%–25%)^9,12^, and Palestine (19.7%)^19^. This variation in prevalence estimates in the Middle Eastern countries may be due to the strictness of tobacco control policies in educational institutions specifically and overall, across populations in general. In this study, vaping-related respiratory and cardiovascular symptoms and signs were significantly more common among vapors than non-vapors for cough, dry mouth, chest pain, palpitations, and shortness of breath.

For high prevalence of an outcome variable, the logistic regression tends to over-estimate the odds ratio as a measure of association between independent variable (s) and the outcome variable^20^. Since in this study, there is moderately high prevalence of vaping in the study sample, we used univariable and multivariable log-binomial regression models to evaluate the association between the independent variable and the outcome variable. The final multivariable log-binomial regression model showed that more male than female students have had significantly and substantially higher prevalence of vaping. This pattern aligns with the findings in other Middle Eastern countries, where societal norms and cultural expectations often associate nicotine use with masculinity^8,17^. Additionally, students registered in literary colleges were more likely to vape compared with those registered in colleges of scientific disciplines. The influence of academic majors may reflect social dynamics and peer norms within these fields, which could contribute to normalizing or discouraging vaping behaviors^11,21,22^.

In this study, participants who believed that vaping is less harmful than cigarette smoking had a significantly higher prevalence of vaping compared with those who either did not believe or were not sure about this notion. This misconception has led to widespread use of e-cigarettes in other parts of the world as well^1,8^, which needs to be corrected with targeted public health education.

The participants in this study, who tended to disbelieve or were unsure that ‘vaping leads to smoking cigarettes’ compared with those who believed this assessment, were significantly more likely to be the vapers. The critics of this view are unintendedly contributing to the widespread use of e-cigarettes^2^. The public health authorities need to make concerted efforts to correct this misconception in the populace, specifically among young adults.

In this study, the participants with the perception of easy accessibility of vaping products had a much higher prevalence of vaping than those who perceived that vaping products were not easily accessible. This finding is consistent with the published literature from other parts of the world that shows a correlation between product accessibility and increased vaping rates among young adults^23^. Regulatory measures that limit accessibility by increasing age restrictions and regulating sales have been effective in countries like New Zealand. Implementing similar regulations in Kuwait could help reduce vaping prevalence among young adults^23^.

Regarding health implications, the prevalences of self-reported short-term respiratory and cardiovascular symptoms were higher among vapors than non-vapors in this study. The higher prevalences of these symptoms among vapors highlight the health impact of vaping on young adults as reported elsewhere^1,24^. These findings are also in parallel with the studies in the US, Canada, and England, where young adult vapers also reported high rates of respiratory distress^6,10^.

### Strengths and limitations

This study has some strengths, including: 1) the study results on university students are directly comparable with the data published from some other countries in the region, which were predominantly collected on university students; and 2) the high response rate (98.2%) in this study tends to flag minimal possible non-participation bias, thus enabling likely generalizability to similar young adults in the general population. A few limitations of this study need to be considered while interpreting the study results: 1) this was a cross-sectional study, and this design has inherent limitations in drawing any causal inference between the identified predictors and the vaping status; 2) the participants were enrolled as a sample of convenience, therefore the generalizability of the findings to other populations needs to be exercised with care; 3) some study variables such as frequency of vape, age at initiation of vape etc., were based on the participants’ recall, therefore bias might have crept in the data; and 4) in the study sample there was a gender imbalance (female to male ratio=3.6:1), the prevalence of vaping might have been somewhat underestimated as vaping practice is more common among males than females. Future studies may consider this aspect at the planning stage to circumvent this pitfall, possibly through stratified sampling. Though we tried to account for the confounding effects of measured confounders, the possibility of residual confounding owing to unknown and/or unmeasured factors cannot be ruled out.

## CONCLUSIONS

In this study, a 15.5% prevalence of current e-cigarette use among young adults in our sample of Kuwaiti students was recorded. In addition to some demographics (i.e. gender, college of study, family income), modifiable factors that include beliefs that ‘vaping is less harmful than cigarettes’, ‘vaping leads to smoking cigarettes’, and accessibility of vaping products were significantly and independently associated with vaping status. The high rates of respiratory symptoms recorded among users underscore the need for targeted public health interventions. If implemented, future studies may evaluate the impact of such interventions.

## Supplementary Material



## Data Availability

The data supporting this research are available from the authors on reasonable request.
